# 3D-printed bredigite scaffolds with ordered arrangement structures promote bone regeneration by inducing macrophage polarization in onlay grafts

**DOI:** 10.1186/s12951-024-02362-2

**Published:** 2024-03-11

**Authors:** Yaowei Xuan, Yibo Guo, Lin Li, Yuzhang, Chenping Zhang, RuiJin, Xuelai Yin, Zhen Zhang

**Affiliations:** 1 Department of Oral and Maxillofacial Surgery, Zhongshan Hospital, Fudan University, Shanghai, 200032 China; 2 Department of Oral & Maxillofacial-Head & Neck Oncology, Shanghai Ninth People’s Hospital, College of Stomatology, National Center for Stomatology, National Clinical Research Center for Oral Diseases, Shanghai Key Laboratory of Stomatology, Shanghai Jiao Tong University School of Medicine, Shanghai Jiao Tong University, Shanghai, 200011 China; 3 State Key Laboratory of Oral & Maxillofacial Reconstruction and Regeneration, Department of Periodontology, School of Stomatology, National Clinical Research Center for Oral Diseases, Shaanxi International Joint Research Center for Oral Diseases, The Fourth Military Medical University, Xi’an, 710032 China

**Keywords:** Onlay graft, Osteoimmunomodulation, Bone regeneration, Bone scaffold, Oral implant

## Abstract

Bone tissue engineering scaffolds may provide a potential strategy for onlay bone grafts for oral implants. For determining the fate of scaffold biomaterials and osteogenesis effects, the host immune response is crucial. In the present study, bredigite (BRT) bioceramic scaffolds with an ordered arrangement structure (BRT-O) and a random morphology (BRT-R) were fabricated. The physicochemical properties of scaffolds were first characterized by scanning electron microscopy, mechanical test and micro-Fourier transform infrared spectroscopy. In addition, their osteogenic and immunomodulatory properties in an onlay grafting model were investigated. In vitro, the BRT-O scaffolds facilitated the macrophage polarization towards a pro-regenerative M2 phenotype, which subsequently facilitated the migration and osteogenic differentiation of bone marrow-derived mesenchymal stem cells. In vivo, an onlay grafting model was successfully established in the cranium of rabbits. In addition, the BRT-O scaffolds grafted on rabbit cranium promoted bone regeneration and CD68 + CD206 + M2 macrophage polarization. In conclusion, the 3D-printed BRT-O scaffold presents as a promising scaffold biomaterial for onlay grafts by regulating the local immune microenvironment.

**Graphical Abstract:**

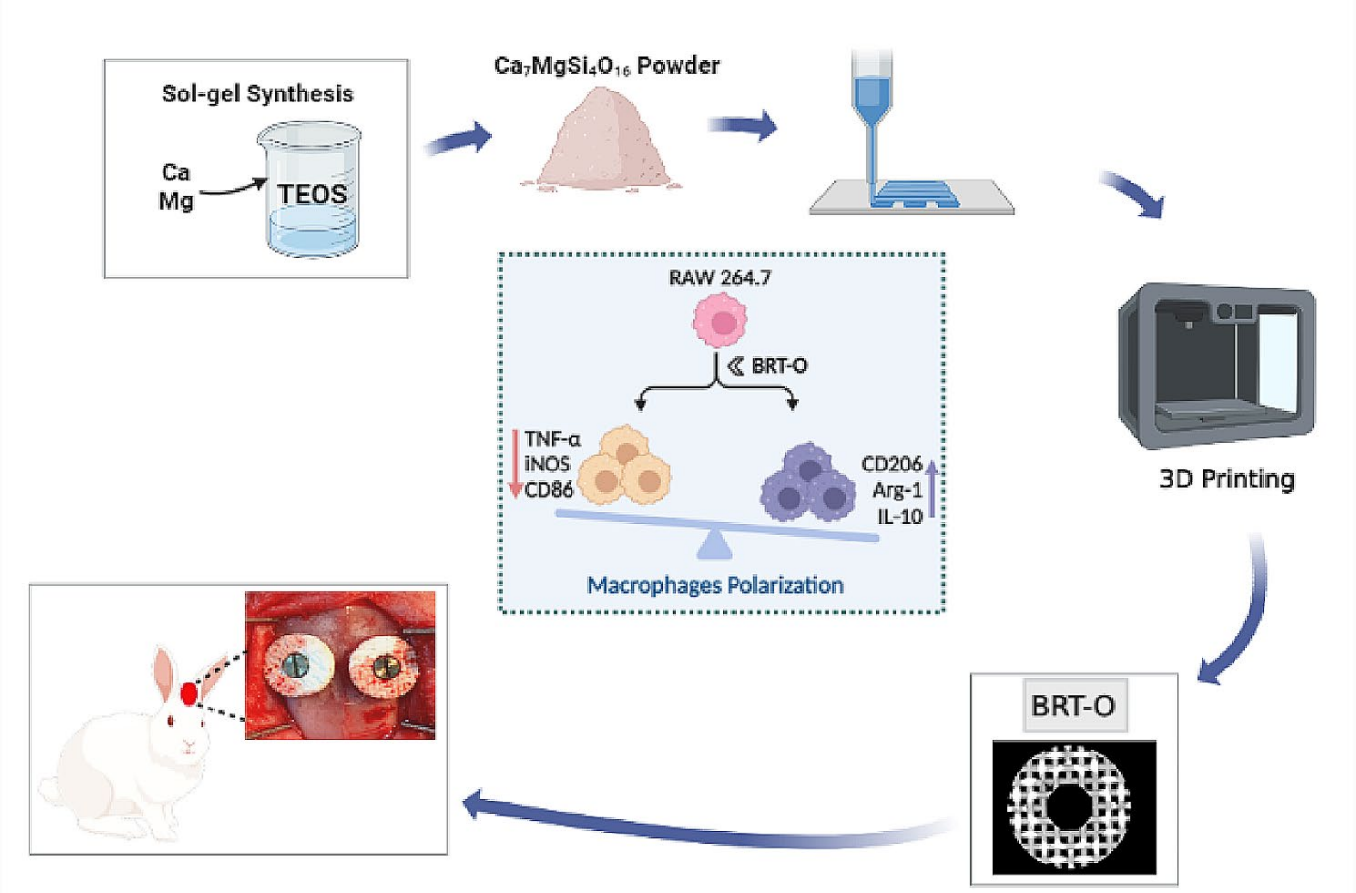

**Supplementary Information:**

The online version contains supplementary material available at 10.1186/s12951-024-02362-2.

## Introduction

Sufficient bone quantity at the site of implant insertion is a crucial prerequisite to enhance the osseointegration and esthetic outcomes of dental implants. However, the alveolar ridge is often deficit due to bone loss, or trauma, infection, or severe periodontitis [1, 2]. The use of autogenous bone grafts, which is also known as onlay bone grafting, is effective for the management of large-sized or severe alveolar bone defects [1, 3–5]. Therefore, autogenous bone grafts have been regarded as the gold standard and first choice for restoring lost alveolar bones. Although autologous bone grafts possess osteogenic, osteoconductive and osteoinductive features, its limitations, such as increased morbidity at the bone harvesting site and unpredictable rate of volumetric bone resorption (18–60%), cannot be ignored [4, 6, 7].

Since the development of bone tissue engineering, various bone regeneration scaffold biomaterials with excellent biological properties, including biocompatibility, osteogenesis, osteoconduction and osteoinduction, have been explored [8, 9]. Bone tissue engineered scaffolds may provide a potential alternative strategy for onlay bone grafts. Present bone regeneration studies have mostly focused on bone defect models, and there are limited studies that involve onlay bone grafts [8–10].

Bioceramic scaffolds with a hierarchical design can mimic the structural and biological characteristics of normal bone tissues, and these have received increasing attention for bone tissue engineering applications [8, 11, 12]. Bredigite (BRT, Ca_7_MgSi_4_O_16_) is a ceramic biomaterial that contains the oxides of various minerals, including calcium (Ca), silicon (Si) and magnesium (Mg). BRT scaffolds have exhibited remarkable effects in inducing osteogenesis and angiogenesis, making it a promising biomaterial for bone tissue engineered scaffolds [12, 13]. In addition, personalized BRT scaffolds can be fabricated using three-dimensional (3D) printing techniques. The 3D printing technique has been widely applied in regenerative medicine due to its unique advantages, such as its rapid, precise and controllable fabrication process [9, 14, 15]. Therefore, it is speculated that using bioceramic materials combined with 3D printing technology may provide a promising alternative strategy for onlay grafts.

Based on the convergence of osteoimmunology and immunomodulation, osteoimmunomodulation has been proposed as the essential ability of biomaterials for modulating bone regeneration [16, 17]. A fundamental principle that underlies this concept is that biomaterials have immunomodulatory properties that are important for generating an osteoimmune environment, which facilitates bone regeneration. Due to its “foreign body” nature, the bone scaffold biomaterial will inevitably alter the local immune microenvironment, and thereby influence the dynamics of bone regeneration [18–20]. Therefore, osteoimmunomodulatory properties must be considered when developing scaffold biomaterials for bone regeneration. After the in vivo grafting of scaffold biomaterials, macrophages are initially recruited to the graft site. Among the various innate immune cells, macrophages play a crucial role in biomaterial-related immune responses [21]. Furthermore, macrophages primarily dictate the long-term immune reactions to biomaterials. Macrophages adapt to different microenvironments by strategically transitioning to either the classically activated M1 phenotype or the M2 phenotype, which respectively mediate inflammation and uphold tissue homeostasis [22, 23]. Previous studies have documented the transition to a different macrophage phenotype as a significant factor in determining the immune response to biomaterials [20, 24]. Since macrophages play a vital role in immune response and bone regeneration, response of macrophages to biomaterial scaffolds have been extensively investigated, with the aim of resolving their osteoimmunomodulatory properties.

The present study aims to evaluate the effects of 3D-printed BRT bioceramic scaffolds in the application of the rabbit cranial onlay grafting model. In addition, the effects of 3D-printed BRT bioceramic scaffolds on the inflammatory reaction, polarization of macrophages, and influence on osteogenesis were evaluated. The present study indicated that the 3D-printed BRT bioceramic scaffold is a promising biomaterial for onlay graft applications and demonstrates osteoimmunomodulatory properties.

## Materials and methods

### Fabrication of the scaffolds

BRT bioceramic scaffolds were fabricated, as previously described [13]. Briefly, Si-, Mg- and Ca-containing BRT (Ca_7_MgSi_4_O_16_) bioceramic powder was synthesized through the sol-gel process, utilizing tetraethoxysilane (TEOS), magnesium dinitrate hexahydrate (Mg(NO_3_)_2_·6H_2_O), and calcium nitrate tetrahydrate (Ca(NO_3_)^2^·4H_2_O). All chemicals were purchased from Sinopharm Chemical Reagent Co. Ltd. (Shanghai, China). The synthetic powder was grounded to particle size by sieving through a 200-mesh screen.

In order to prepare the 3D printer bioinks for the BRT scaffolds, 0.15 g of sodium alginate powder and 5.0 g of BRT powder were added to 3.0 g of Pluronic F-127 (20.0 wt%). Then, the ink was extruded through a nozzle (inner diameter: 0.22 mm), which was controlled using the Nano-Plotter^™^ software (GeSiM, Radeberg, Germany). Afterwards, the 3D printed primary BRT scaffolds were calcined for three hours (1,350 °C, heating rate: 2 °C min^− 1^). According to the standard tessellation language file of the 3D models, BRT scaffolds with an ordered arrangement structure (BRT-O) and BRT scaffolds with a random morphology (BRT-R) were fabricated by laying patterns of filaments (0°/90°). Then, the fabricated scaffolds were sterilized by UV irradiation for further in vitro and in vivo evaluation.

### Scaffolds characterization

The surface morphology of the BRT scaffolds was analyzed by scanning electron microscopy (SEM; Hitachi S-4800, Tokyo, Japan) at an accelerating voltage of 20 kV. The chemical components and spatial matrix distribution were evaluated by micro-Fourier transform infrared spectroscopy (Bruker, USA). The mechanical properties of BRT scaffolds were analyzed using a universal mechanical testing machine (INSTRON 5566, Norwood, MA, USA), with a movement speed of 1.0 mm min^− 1^. In addition, the ion release, pH value, and weight change of the scaffold degradation were evaluated in vitro by placing these scaffolds in a Tris-HCl solution (pH 7.40) for different periods. Then, the samples were incubated in a shaking incubator at 37 °C. Afterwards, the solution was collected and refreshed on day 3, 7, 14, 28 and 35. Inductively coupled plasma atomic emission spectrometry (ICP-AES; Varian Co., USA) was used to evaluate the concentration of Ca, Mg and Si ions, and a pH meter (Metrohm, Germany) was used to monitor the pH value of the solutions. For accurate weight measurements, the scaffolds were removed and dried at 120 °C for 24 h before weighing using a digital scale. All experiments were carried out in triplicate.

### Evaluation of the effects of scaffolds on macrophage polarization

Murine macrophage RAW264.7 cells were obtained from the Typical Culture Preservation Commission Cell Bank, Chinese Academy of Sciences (Shanghai, China). These cells were cultured in Dulbecco’s Modified Eagle Medium (DMEM, Gibco), which contained 10% FBS and 1% penicillin/streptomycin, in a 5% CO_2_ incubator at 37 °C. Then, the RAW264.7 cells were cultured in 6-well plates with different scaffolds for the indicated time period.

The relative levels of iNOS and arginase 1 (Arg-1) gene mRNA transcripts to the control GAPDH in different groups of RAW264.7 cells were quantified by quantitative real-time polymerase chain reaction (qRT-PCR) using specific primers (Table S1). Enzyme-linked immunosorbent assay (ELISA; Dakewe Bioengineering, China) was used to determine the concentration of cytokines (IL-10 and TNF-α) in the supernatants, according to manufacturer’s instructions. Furthermore, the effect of scaffolds on the polarization of macrophages was analyzed by flow cytometry (BD Accuri C6, USA) using antibodies against CD86 (1:50 dilution; BioLegend, USA) and CD206 (1:400 dilution; BioLegend, USA). In addition, immunofluorescence staining was performed after 24 hours of culture. Briefly, primary antibodies against CD68, CD206 and iNOS (1:1,000 dilution; Abcam, USA) were dropped onto coverslips, and incubated overnight at 4°C. Then, secondary antibodies Alexa Fluor 488 goat anti-mouse IgG (1:200, Abcam, USA) and Alexa Fluor 594 goat anti-rabbit IgG (1:200, Abcam, USA) were applied to react with the primary antibodies for one hour at room temperature, followed by nuclear staining with 4’,6-diamidino-2-phenylindole (DAPI) for five minutes. Afterwards, the images were captured and visualized using laser scanning confocal microscopy (LSCM; Olympus, Japan). SEM was performed to observe the morphology of the RAW 264.7 cells seeded on different scaffolds for one day.

### Assessment of the effects of polarized macrophages on the proliferation, migration and differentiation of bone marrow-derived mesenchymal stem cells (BMSCSs) in vitro

In order to evaluate the macrophages in response to the scaffold’s impact on BMSCSs, supernatant samples were collected from wells that contained RAW264.7 cells seeded on scaffolds after three days of culture, in order to prepare a conditioned medium (CM). Then, the BMSCSs from the femurs and tibias of male C57BL/6 N mice (aged 6–8 weeks) were isolated, cultured and identified using a well-established technique, as previously described [25]. Briefly, BMSCSs were cultured in 96-well plates, and treated with a mixture of DMEM and CM, at a ratio of 1:1. BMSCSs cultured with unconditioned medium were used as the control.

After 1, 3 and 5 days of culture, the proliferation of BMSCSs was evaluated by CCK-8 assay. Wound scratch assay and transwell assay were performed to evaluate the migration of BMSCSs. For the wound scratch assay, BMSCSs were seeded in six-well plates with the basal medium. When the cell confluency reached at 90–100%, a scratch was made on the cell layer using the head of a 200-µL pipette tip (Axygen, USA). Then, serum-free CM was added, and the cells were further incubated for 12 and 24 h. The cells without adding CM were used as a control group. Afterwards, the cell migration was observed under an optical microscope (Olympus, Japan), and the healing area was calculated using the ImageJ software (NIH, USA). In addition, Boyden chambers were applied for the transwell analysis. The BMSCSs were placed in the upper chamber with basal medium, while the RAW264.7 cells were seeded on different scaffolds in the lower chambers. After culturing for 24 h, the penetrating cells were fixed with 4% paraformaldehyde, stained with crystal violet solution, and analyzed under an optical microscope (Olympus, Japan).

After culturing with the supernatants for seven days, osteogenic markers bone morphogenetic protein 2 (BMP2) and RUNX2 in BMSCSs were evaluated by qRT-PCR and western blot. For the qRT-PCR, the primers used are presented in Table S1. For the western blot, anti-RUNX2 (1:1,000; ab23981, Abcam) was used as the primary antibody, and conjugated horseradish peroxidase was used as the secondary antibody. The formation of calcium nodules was evaluated on the 21st day using Alizarin red S (ARS). The rinsed BMSCSs were fixed in 4% paraformaldehyde, and stained using 2% ARS solution in room temperature for 20 min. The stained calcium deposits were dissolved in 10% cetylpyridinium chloride (Sigma-Aldrich, USA) for 15 min, and the dye release was quantified by spectrophotometry at 562 nm (Thermo Fisher Scientific, USA).

### In vivo onlay graft regeneration

All animal procedures were approved by the Animal Experimental Ethics Committee of the Ninth People’s Hospital Affiliated with Shanghai Jiao Tong University School of Medicine. The study design followed the ARRIVE (Animal Research: Reporting of In Vivo Experiments) guidelines.

#### Animal model for the onlay graft

A total of 27 New Zealand white male rabbits (weighing 2.8 ± 0.2 kg) were purchased from the Experimental Animal Centre of the Ninth People’s Hospital, China. All animals were kept under standard conditions (controlled temperature at 22 ± 2 °C, humidity at 55 ± 5%, light/dark cycle of 12/12 hours) with water and food *ad libitum*. These animals were administered with general anesthesia by intravenously injecting pentobarbital sodium (30 mg/kg, Sigma), followed by shaving of the cranium hair and disinfection. Then, a longitudinal incision was performed along the midline in the cranium, and the soft tissue flap was elevated to expose the cranial region. After preparation of the recipient site by decortication, the scaffolds were grafted in the corresponding bone bed on both sides of the midline, and fixed using a titanium screw. For the autologous bone graft group, the circular bone blocks (φ: 8 mm) harvested from the cranium with a dental trephine bur were fixed alongside the defects. Then, the incision was closed with suture by layers. Accordingly, the rabbits were allocated into the following three groups: (1) autograft group; (2) BRT-R group; (3) BRT-O group. These rabbits were routinely housed and fed under standardized conditions, and euthanized at 2, 6, or 16 weeks by overdose of intravenous sodium pentobarbital (150 mg/kg), postoperatively. The cranial specimens were harvested for further evaluation.

#### Micro-CT analysis

The samples were fixed with 4% paraformaldehyde, and evaluated using the micro-computed tomography system (micro-CT; Scanco Medical, Bassersdorf, Switzerland) with the following parameters: 70 kV voltage, 114 mA current, and 700 ms integration time. The obtained images were analyzed using the µ-CT 80 system software for 3D construction. Cylinders with a diameter of 8 mm and a height of 1 mm were selected as the volume of interest (VOI). Then, the bone mineral density (BMD) and bone volume/tissue volume (BV/TV) were calculated for the quantitative analysis of the bone regeneration within each VOI.

#### Histological and immunohistochemical evaluation

For the histological evaluation, a part of the samples was dehydrated and embedded into polymethyl methacrylate (PMMA). Without decalcification, the tissue blocks were cut into sections (thickness: 200 μm) using a hard tissue microtome (Leica, Germany), and sequentially polished to a final thickness of 25 μm. Then, these sections were stained with the toluidine blue staining solution for new bone regeneration analysis. The other part of the samples was decalcified with 10% ethylenediaminetetraacetic acid, and prepared into 10-µm-thick sections. The histological evaluation of the newly formed bone and remnant scaffold was performed using hematoxylin and eosin (H&E) staining. Three fields of view were randomly selected for each slice. The images were observed under a microscope (Olympus dp51), and captured using a digital camera (DXM1200). The Image-Pro Plus software (Media Cybernetics, Inc.) was used to compare the osteogenic properties of the different scaffold materials.

For the immunohistochemical staining, the two-week postoperative specimens were dewaxed and incubated with primary antibodies against RUNX2 (Abcam, USA) for the osteogenic marker, CD68 (Abcam, USA) for the pan-macrophage marker, CD206 (Abcam, USA) for the M2 marker, and iNOS (Abcam, USA) for the M1 marker at 1:100 dilution overnight at 4 °C, followed by incubation with the secondary antibody. Then, the stained sections were viewed under an optical microscope (Leica DMI 6000B Microsystems, Germany), and the proportion of positive cells was calculated at 40× magnification.

### Statistical analysis

All statistical analyses were performed using GraphPad Prism 8.0 (GraphPad Software Inc., USA) through analysis of variance with Tukey’s post-hoc test. The data were presented as mean ± standard deviation. A *p*-value of < 0.05 was considered statistically significant.

## Results

### Scaffolds characterization

**Fig. 1:**
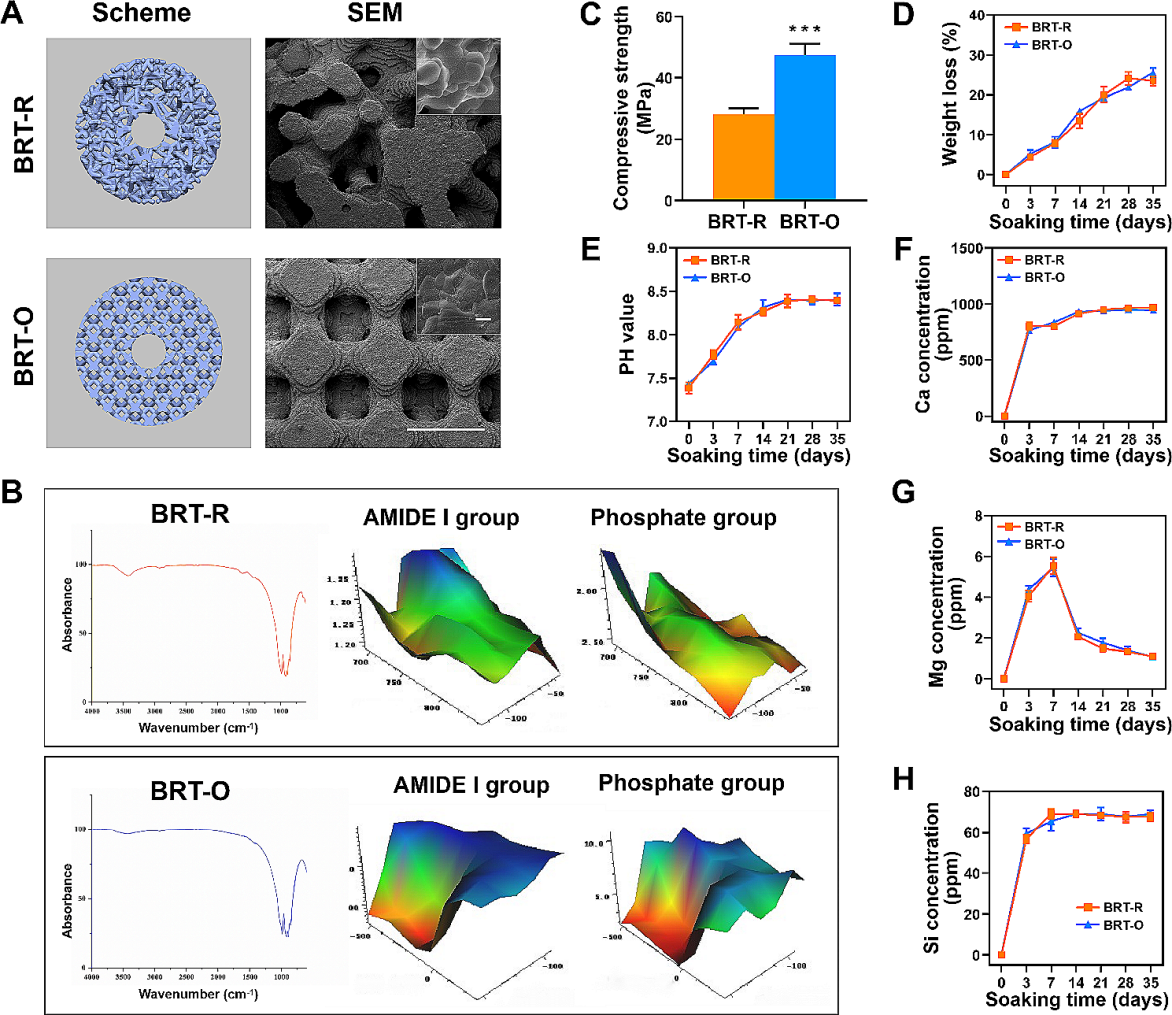
The characterization of bredigite scaffolds. (**A**) The schematic and scanning electron microscopy images of the BRT-R and BRT-O scaffolds. (**B**) The micro-FTIR spectra and mappings of the scaffolds. (**C**) The compressive strength of the scaffolds. (**D**) Weight loss (%) of the scaffolds after soaking in buffer for different time periods. (**E**) The pH value change after soaking the scaffolds at different time points. The (**F**) Ca, (**G**) Mg and (**H**) Si concentration in Tris buffer at different time points. The data were expressed as mean ± standard deviation (SD) (*n* = 3). ^***^p < 0.001 vs. BRT-R. Scale bar: A, 1 mm and 5 μm

The 3D model and SEM images of the scaffolds are presented in Fig. 1A. The scaffolds were 8 mm in diameter and 2 mm in height. The SEM images revealed the macroporous structures for both types of scaffolds, which were likely to facilitate the ingrowth of blood vessels, nutrients diffusion, and tissue regeneration. In addition, the BRT-O scaffold revealed a specific uniform and ordered arrangement of microstructure, when compared to the BRT-R scaffold (Fig. 1A). The FTIR analysis indicated the chemical components and spatial matrix distribution of the scaffolds at the micron level (Fig. 1B). Both scaffolds had typical peaks, indicating an identical similar chemical composition. The FTIR maps demonstrated a consistent spatial distribution in the amide I and phosphate groups in both scaffolds. Typical vibration bands of phosphate were identified in both groups. For the mechanical analysis, the compressive strength of the BRT-O scaffold was 1.69 times higher than that of the BRT-R scaffold (Fig. 1C). The analysis of degradation and ion release behavior indicated that along with the increase in soaking time, both scaffolds exhibited a sustained weight loss (Fig. 1D). The pH values of the Tris solution gradually increased up to 14 days, and these subsequently remained stable in both groups (Fig. 1E). Meanwhile, both scaffolds exhibited a similar ion release profile for Ca, Si and Mg ions (Fig. 1F and H). There was no significant difference in BRT-O and BRT-R scaffolds, in terms of pH, degradation, and ion release behavior.

### BRT-O scaffolds induce M2 macrophage polarization in vitro

**Fig. 2:**
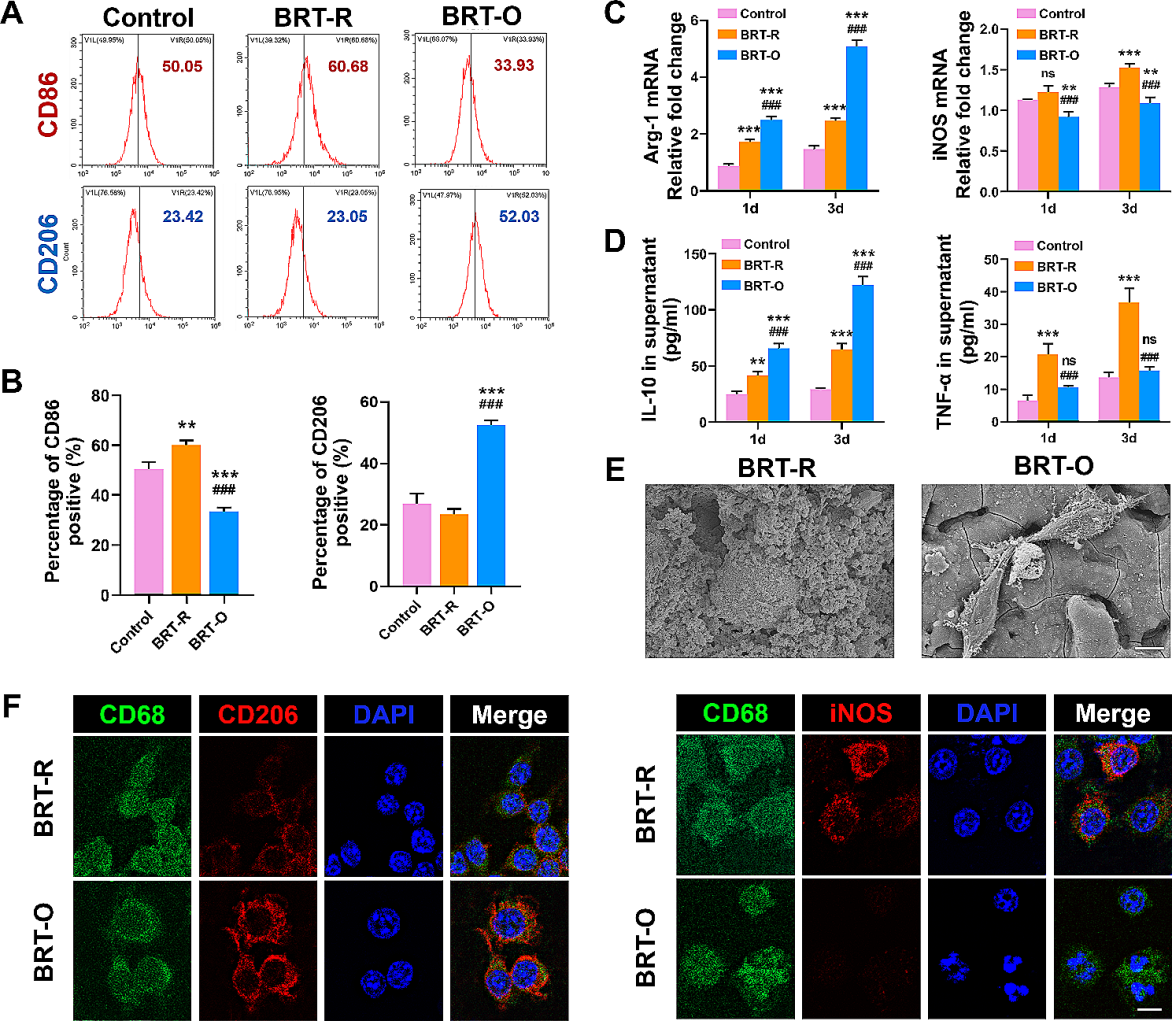
Scaffolds polarized the macrophage phenotypes in vitro. (**A** and **B**) Flow cytometry analysis of RAW264.7 cells that were seeded on different scaffolds for three days. (**C**) The qRT-PCR analysis of the macrophage polarization-related gene expression at day three. (**D**) The enzyme-linked immunosorbent assay analysis of cytokines in the supernatants of RAW264.7 cells cultured on different scaffolds on day three. (**E**) The morphology of macrophages seeded on different scaffolds visualized by scanning electron microscopy. (**F**) The immunofluorescent staining of macrophages seeded on different scaffolds on day three with CD68 (green), CD206 or iNOS (red), and nuclei (blue). The data were expressed as mean ± standard deviation (SD) (*n* = 3). The cells without scaffolds were set as a control group. Ns, no significance; ^**^p < 0.01 vs. the control group; ^***^p < 0.001 vs. the control group; ^###^*p* < 0.01 vs. the BRT-R group. Scale bars: E, 5 μm; F, 50 μm

Flow cytometry analysis was performed to evaluate the effect of scaffolds on the polarization of macrophages. The results indicated that RAW264.7 cells seeded on BRT-O scaffolds decreased the CD86 expression, but increased the CD206 expression, when compared to the BRT-R scaffold (Fig. 2A and B). In addition, the mRNA expression levels of Arg-1 were higher and the levels of iNOS were significantly lower in the BRT-O group, when compared to the other groups (Fig. 2C). A similar trend was observed in the ELISA experiments, in terms of the cytokine levels of TNF-α and IL-10 (Fig. 2D). The SEM analysis revealed that macrophages on the BRT-O scaffolds exhibited a highly branched and elongated morphology, when compared to the BRT-R scaffolds (Fig. 2E). Furthermore, the immunofluorescence staining indicated that macrophages on the BRT-O scaffold expressed the CD206 and CD68 M2 macrophage markers, while macrophages on BRT-R scaffolds expressed the CD68 and iNOS M1 macrophage markers (Fig. 2F). These findings suggest that BRT-O scaffolds facilitate the macrophage polarization towards the M2 phenotype.

### Polarized macrophages promote BMSCS migration and osteogenic differentiation in vitro

**Fig. 3:**
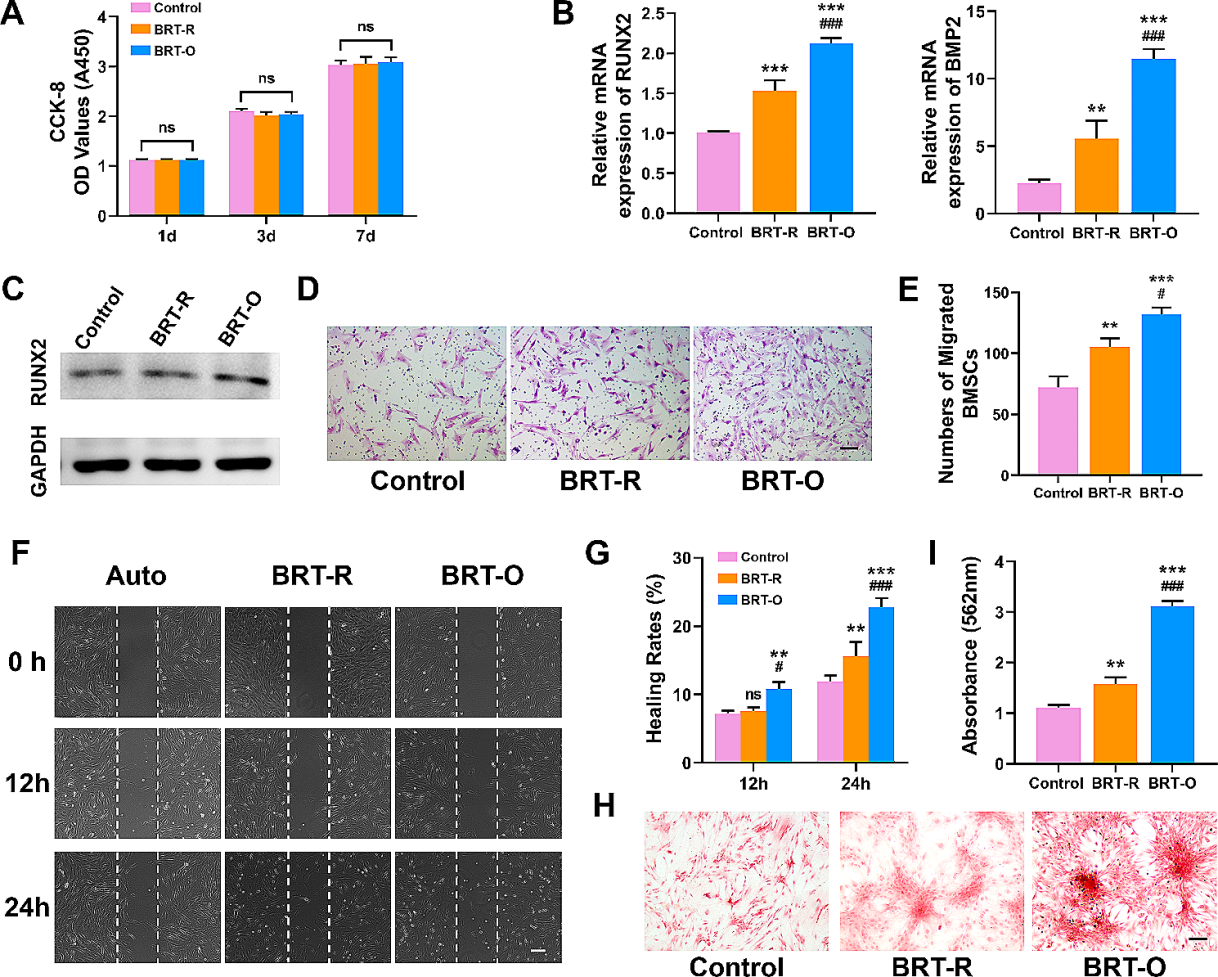
Polarized macrophages by BRT-O scaffolds promoted the osteogenic differentiation of BMSCSs in vitro. (**A**) CCK-8 analysis of BMSCSs after 1, 3 and 7 days of co-culture with different conditioned media (CM). (**B**) The qRT-PCR analysis of the relative mRNA expression levels of RUNX2 and BMP2 in BMSCSs on day seven. (**C**) Western blot results for the RUNX2 expression levels in BMSCSs on day seven. (**D**) The transwell assay of BMSCSs induced by different CM. (**E**) The quantification analysis of cell migration. (**F**) Representative images of the wound healing capacities of BMSCSs after co-culture with different CM for the indicated time periods. (**G**) Quantitative analysis of the wound healing. (**H**) ARS staining of BMSCSs on day 21. (**I**) Semi-quantification analysis of ARS. The data were expressed as mean ± standard deviation (SD) (*n* = 3). Ns, no significance; ^**^p < 0.01 vs. the control group; ^***^p < 0.001 vs. the control group; ^#^*p* < 0.05 vs. the BRT-R group; ^###^*p* < 0.01 vs. the BRT-R group. Scale bars: D, 100 μm; F, 200 μm; H, 100 μm

The effects of polarized macrophages through the scaffolds on the proliferation, migration and osteogenic differentiation of BMSCSs were further evaluated. The CCK-8 results revealed that there was no significant difference in the proliferative activity of cells among the groups (Fig. 3A). Furthermore, the osteogenic differentiation-related gene RUNX2 mRNA expression was significantly upregulated in the BRT-O group, when compared to the other two groups (Fig. 3B). Moreover, the western blot analysis revealed that the protein levels of RUNX2 exhibited a similar trend (Fig. 3C). The wound scratch assay and transwell assay indicated that the migration of BMSCSs was promoted by macrophages seeded onto the BRT-O scaffolds (Fig. 3D and G). Furthermore, the ARS results indicated that the size and quantity of mineral nodules were remarkably larger in the BRT-O group, when compared to the BRT-R group (Fig. 3H and I). In summary, it was demonstrated that M2 macrophages are polarized by BRT-O scaffolds, in order to facilitate the migration and osteogenic differentiation of BMSCSs.

### BRT-O scaffold promotes bone regeneration in onlay grafting

**Fig. 4:**
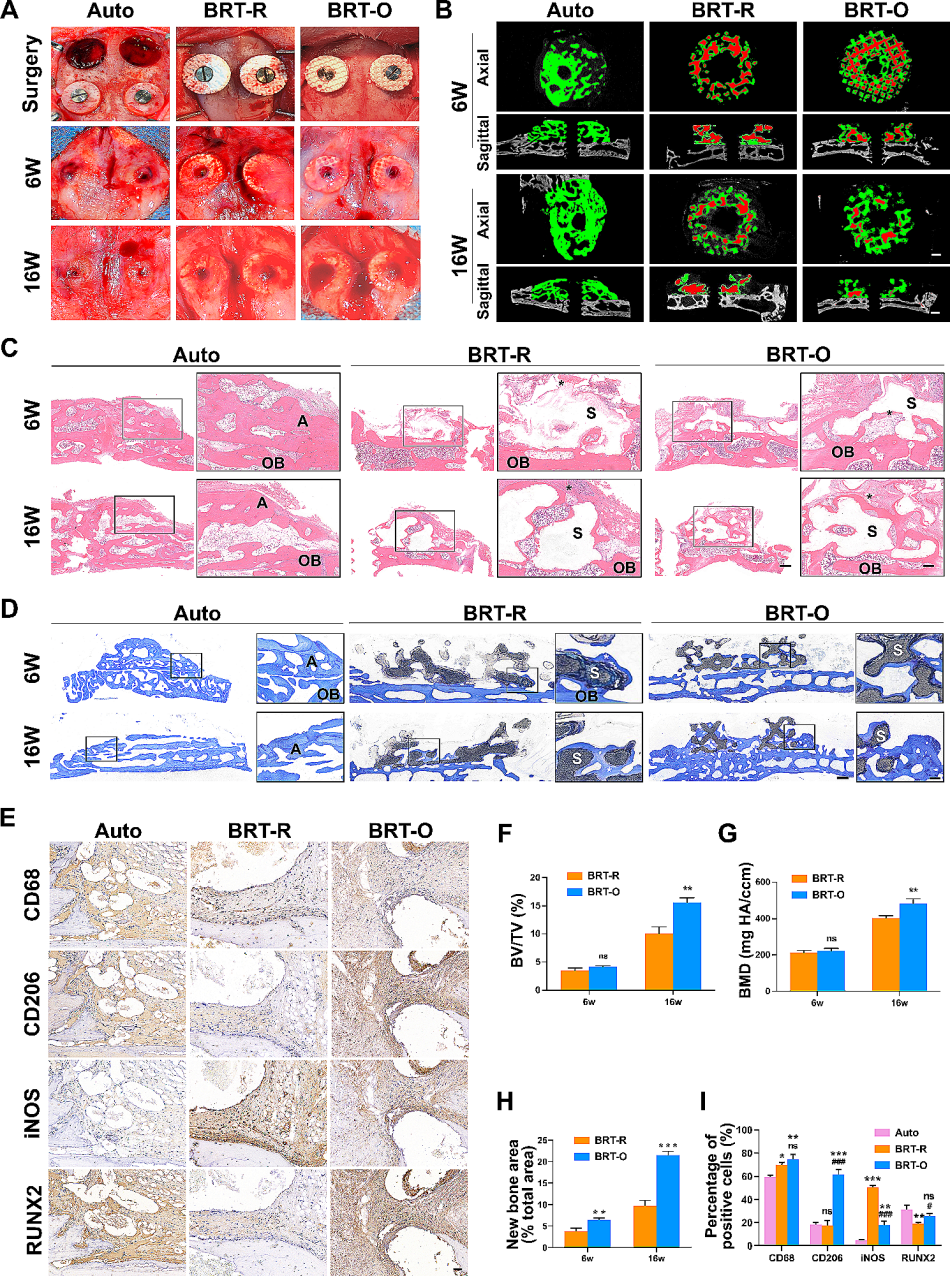
The BRT-O scaffold promoted bone regeneration in the onlay grafting model of rabbit cranium. (**A**) Images of the surgical procedure, and specimens of the different groups at six and 16 weeks after surgery. (**B**) Representative micro-CT images of different groups at six and 16 weeks, post-surgery. Representative images for the (**C**) H&E and (**D**) toluidine blue staining of samples collected at six and 16 weeks, post-surgery (A, auto graft; S, scaffold; ^*^, new bone; OB, old bone). (**E**) Immunohistochemical staining for RUNX2 and macrophage polarization pan marker CD68, M1 marker iNOS, and M2 marker CD206 in the onlay grafting area. Quantitative results for (**F**) BV/TV and (**G**) BMD by micro-CT. (**H**) Quantitative results for the new bone (%) in the scaffold area. (**I**) Quantification of positively stained cells. The data were expressed as mean ± standard deviation (SD) (*n* = 3). Ns, no significance; ^**^p < 0.01 vs. the control group; ^***^p < 0.001 vs. the control group; ^#^*p* < 0.05 vs. the BRT-R group; ^###^*p* < 0.01 vs. the BRT-R group. Scale bars: B, 1 mm; E, 500 μm and 200 μm; F, 300 μm and 200 μm; H, 500 μm

A rabbit cranial onlay grafting model was successfully established to investigate the osteogenic effects of BRT scaffolds. The surgical procedures are presented in Fig. 4A. During the entire experiment, no animal deaths or obvious signs of infection were observed. Furthermore, the macroscopic images revealed no signs of necrosis or obvious inflammation in any of the specimens (Fig. 4A). Moreover, the bone volume of the autogenous onlay graft decreased with time, and new tissue was regenerated through the porous structure of both scaffolds.

The micro-CT images of the onlay graft area in the rabbit calvarial bone at six and 16 weeks post-implantation are presented in Fig. 4B. The reconstructed images show that the autogenous bone residues decreased with time, which is consistent with the macroscopic observation. In addition, the transverse and sagittal sections of the micro-CT images revealed that the residual scaffold material (red) decreased with time, and was accompanied by the ingrowth regeneration of new bone tissues (green), in both the BRT-R and BRT-O groups. The quantitative analysis of the micro-CT is presented in Fig. 4F and G. At week six, no significant difference was found in both groups, in terms of the BMD of newly formed tissues and BV/TV values. At week 16, the BMD measurement in the BRT-O group (482.2 ± 26.81 mg HA ccm^− 1^) was 1.20 times higher than that of the BRT-R group (402.2 ± 13.41 mg HA ccm^− 1^). A similar trend was observed for the BV/TV value (Fig. 4G).

The histological evaluation conducted by H&E staining for decalcified sections and toluidine blue staining for undecalcified sections revealed the evident bone resorption of autogenous bone, which mainly occurred at the margin of the bone blocks (Fig. 4C and D). Meanwhile, the regeneration of new bone was observed on the surface, and this extended to the pores in both the BRT-R and BRT-O groups. At week 16, a relatively mature formed bone with abundant cuboidal-shaped osteoblasts and a Haversian canal-like structure was observed in the BRT-O group (Fig. 4C and D). The quantitative analysis indicated that the new bone area was significantly greater in the BRT-O group (49.1 ± 7.69%), when compared to the BRT-R group (12.2 ± 3.94%) (*p* < 0.01) at week 16 (Fig. 4H).

The osteoimmunomodulatory properties of the BRT scaffold in the early period of onlay grafting were evaluated by immunohistochemical staining (Fig. 4E). It was observed that the percentage of RUNX2 positive cells was significantly higher in the auto group and BRT-O group, when compared to the BRT-R group. Meanwhile, the number of CD68+ (94.5 ± 3.7) and CD206+ (67.6 ± 4.7) M2 macrophages were high, while the number of iNOS + M1 macrophages was relatively low (19.3 ± 1.1) in the BRT-O group after two weeks (Fig. 4I). In the auto group, the positive staining of pan-macrophage marker CD68 was also identified. However, the number of was evidently lower, when compared to that in the BRT-R and BRT-O groups (*p* < 0.05). In addition, relatively low numbers of CD206 + M2 macrophages or iNOS + M1 macrophages were observed in the auto group (Fig. 4I).

## Discussion

In the present study, an animal model of rabbit calvarial onlay bone grafting was successfully established to evaluate the regenerative potential of 3D-printed BRT bioceramic scaffolds in the repair of bone defects. In addition, the effect of 3D-printed BRT bioceramic scaffolds on the inflammatory reaction and polarization of macrophages and osteogenesis was investigated. The present study revealed that 3D-printed BRT scaffolds with an aligned structure can polarize macrophages to the pro-regenerative M2 phenotype. This improves the migration and osteogenic proliferation of BMSCSs, promoting bone regeneration in the onlay bone grafting model (Schematic 1). The 3D-printed bioceramic BRT-O demonstrated a promising potential as osteo-immunomodulatory bone scaffold biomaterials for onlay bone graft applications.

At present, unpredictable absorption of the autologous bone block remains as the main drawback in autogenous onlay bone grafting for oral and maxillofacial tissues, which may lead to insufficient bone volume, indicating the need for another bone augmentation procedure [1, 5, 26]. Consequently, reducing the bone resorption of onlay autologous bone grafts has become a research hotspot in the field of contemporary oral implants. In clinic, the key strategies to reduce the resorption of onlay autologous bones include the following: harvesting the bone block from the donor site with an intramembranous bone formation origin (such as the ramus, lateral oblique line, and mental region of the mandible), decorticated preparation of the recipient area, rigid internal fixation of the bone block, tension-free sutures, combining with guided bone regeneration procedures, and so on [1, 3–5, 7]. In the present study, the compression test confirmed the sufficient mechanical strength of BRT scaffolds to withstand the pressure of the surrounding soft tissues. In addition, decorticated preparation of the recipient area, fixation of scaffolds with titanium screws, and tension-free sutures were performed to ensure the promotion of osteogenic effects.

In clinic, implant sites that require bone augmentation procedures are usually irregular in shape. Thus, it remains challenging to precisely reconstruct irregular bone defects using traditional autologous bone grafts. Recently, 3D printing technology has rapidly evolved for tissue regeneration applications [27]. The 3D printing technology offers a huge advantage, in terms of meeting individual implant requirements. Furthermore, through 3D printing technology, bone scaffolds can be fabricated in various geomatical shapes, according to individual needs, in order to accommodate complex bone defects, dimensions, and intricated internal morphologies. Accordingly, 3D printing technology combined with tissue engineering scaffolds and digital design is likely to have more potential applications in onlay bone grafts for oral implantology.

Conventional bone scaffold biomaterials mainly concentrate in optimizing its capacity for osteoinduction, while the concentration of immune responses elicited by these has received relatively less attention [17]. However, immune and skeletal systems widely interplay, leading to a concept known as osteoimmunology [16, 17, 28]. Recently, researchers have focused more on the osteoimmunomodulatory properties of bone scaffold biomaterials, aiming to regulate immune reactions, and promote bone regeneration [20, 29–31]. After biomaterials grafting in vivo, macrophages are the earliest cells recruited at the grafting site [25, 32]. Furthermore, macrophages predominantly determine the long-term immune reactions to biomaterials [32–34]. Moreover, macrophages can tactically switch to different phenotypes, in order to adapt to variable microenvironments. Previous investigations have indicated that the phenotype of macrophages can affect the outcomes of bone regeneration after grafting bone scaffolds. Tuning macrophages toward the pro-regenerative M2 phenotype can evidently facilitate bone regeneration, which is consistent with the findings of the present study [25, 32–34]. Future investigations should focus on the underlying mechanisms of macrophage regulation for designing bone scaffolds with immunomodulation strategies in the field of regenerative medicine.

Bone scaffold biomaterials differ, in terms of topological cues, chemistry, porosities, released bioactive ions, etc. These may have different impacts on the local microenvironment for the recruitment and differentiation of host cells [35–38]. Previous studies have demonstrated that the surface topological cues of biomaterials can influence the immune microenvironment [20, 30, 39–42]. In the present study, the 3D-printed BRT scaffold with an ordered arrangement structure affected the immune microenvironment by modulating the macrophage polarization towards the pro-regenerative M2 phenotype. These findings are consistent with the reports of previous studies, suggesting that modifying the topological cues is a promising strategy for immunomodulation and bone regeneration [37, 42, 43]. In addition, inorganic bioceramics have great potential for immunomodulatory activity in tissue regeneration [20, 33, 44]. In the present study, the BRT bioceramics polarized the macrophages toward M2 cells, both in vitro and in vivo, and determined the osteogenesis outcomes. These findings advocate the concept, in which the release of inorganic bioactive ions, such as Zn, Mg, Mo and Sr ions, can modulate the immune microenvironment by promoting the macrophage polarization to the M2 phenotype [20, 33, 34, 44, 45]. Therefore, it is reasonable to speculate that ions released from BRT may modulate the immune response of macrophages. In the future, the investigators will determine the role of individual ions in BRT scaffolds, in terms of regulating the immune microenvironment during the bone regeneration process.

Although the potential application of 3D printed BRT-O scaffolds in onlay grafts was proposed, using bone scaffolds alone still has certain limitations in bone regeneration outcomes. Further strategies for optimizing bone scaffold materials may focus on combining stem cells, growth factors, and 3D bioprinting technology, and these are expected to lead to better clinical outcomes. In addition, local immune responses following biomaterial grafting may not be limited to the activation of macrophages. Thus, there is a need to conduct further studies on other types of immune cells, such as neutrophils and T cells, and its contributions to the process of bone regeneration at the molecular level.

## Conclusion

In summary, the present study indicated that 3D printed BRT scaffolds with an ordered arrangement structure induces macrophage polarization towards the pro-regenerative M2 phenotype. In addition, macrophages polarized by BRT-O scaffolds can enhance the migration of BMSCSs, and facilitate osteogenic differentiation. More importantly, BRT-O scaffolds can adequately improve the proportion of pro-regenerative macrophages and bone regeneration in an onlay grafting model of rabbit cranium. These findings highlight the potential applications of 3D printed BRT scaffolds with an ordered arrangement structure in the bone regeneration of onlay bone grafts from an immunomodulatory perspective.

### Electronic supplementary material

Below is the link to the electronic supplementary material.

Supplementary Material 1
