# Supplementary material for: 3D-printed bredigite scaffolds with ordered arrangement structures promote bone regeneration by inducing macrophage polarization in onlay grafts
Source: J Nanobiotechnology. 2024 Mar 11;22:102. doi: 10.1186/s12951-024-02362-2 (PMC10926610; doi:10.1186/s12951-024-02362-2)
Supplement: Supplementary file 1 — Supplementary Material 1 [file 12951_2024_2362_MOESM1_ESM.docx]

**Table S1. Primer sequences for qRT-PCR**

| Gene name | Forward (5’ – 3’) | Reverse (5’ – 3’) |
| --- | --- | --- |
| *Gapdh* | ATGATTCTACCCACGGCAAG | CTGGAAGATGGTGATGGGTT |
| *Arg1* | CGCCTCAAATCCAGCTGTAAG | GGGCCACAATCCAGTCGTT |
| *Nos2* | TGGTGAAAGTGGTGTTCT | TTCCCTGTCTCAGTAGCA |
| *Bmp2* | ATCCAGTCTTGCCGCCTCCAG | CTTCGCCTCCTCCTCCTTCTCC |
| *Runx2* | CCTTCAAGGTTGTAGCCCTC | GGAGTAGTTCTCATCATTCCCG |
